# The Battle for Survival: The Role of RNA Non-Canonical Tails in the Virus–Host Interaction

**DOI:** 10.3390/metabo13091009

**Published:** 2023-09-13

**Authors:** Xianghui Wen, Ahsan Irshad, Hua Jin

**Affiliations:** Key Laboratory of Molecular Medicine and Biotherapy, School of Life Science, Beijing Institute of Technology, No. 5 South Zhongguancun Street, Beijing 100081, China; 3120201420@bit.edu.cn (X.W.); ahsanaari@gmail.com (A.I.)

**Keywords:** mixed tail, uridylation, TENT4, TUT4/7, virus, antiviral therapy

## Abstract

Terminal nucleotidyltransferases (TENTs) could generate a ‘mixed tail’ or ‘U-rich tail’ consisting of different nucleotides at the 3′ end of RNA by non-templated nucleotide addition to protect or degrade cellular messenger RNA. Recently, there has been increasing evidence that the decoration of virus RNA terminus with a mixed tail or U-rich tail is a critical way to affect viral RNA stability in virus-infected cells. This paper first briefly introduces the cellular function of the TENT family and non-canonical tails, then comprehensively reviews their roles in virus invasion and antiviral immunity, as well as the significance of the TENT family in antiviral therapy. This review will contribute to understanding the role and mechanism of non-canonical RNA tailing in survival competition between the virus and host.

## 1. Introduction

**Figure 1:**
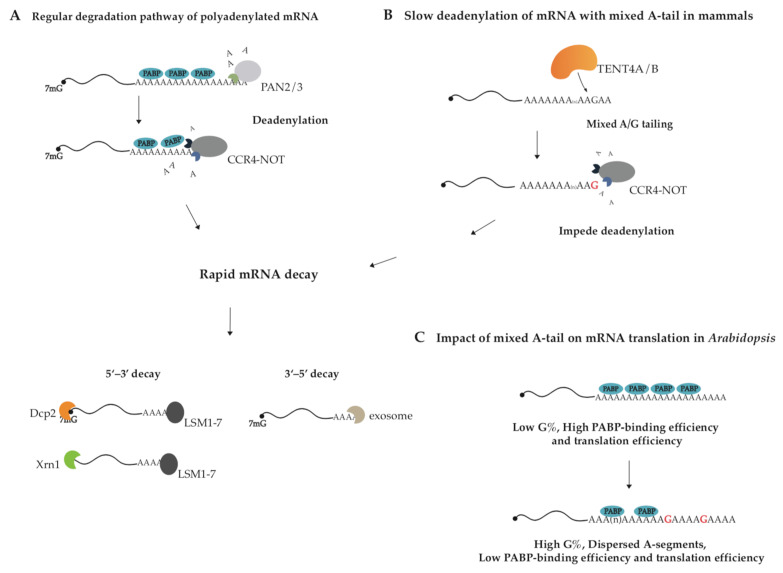
The proposed model for the action mechanism of mixed tailing. (**A**,**B**) TENT4A/4B stabilizes mRNA by generating mixed A/G tailing in human cells. PAN2/3 shortens poly(A) tail to 110 nt, and CCR4-NOT removes the remaining A-residues. TENT4A/4B decorates poly(A) tail with guanosine residues. Compared with pure poly(A) tail, mixed A/G tail is more resistant to CCR4-NOT-complex-mediated deadenylation since CCR4-NOT sheds once it encounters G-residue. After deadenylation and decapping, all mRNAs are degraded from both 5′ and 3′ ends. (**C**) Mixed A/G tails regulate translation efficiency in *Arabidopsis*. Guanosines are supposed to divide the poly(A) tail into interspersed A-segments, thereby reducing binding efficiency between PABP and A-tail and translation efficiency of *Arabidopsis* mRNA.

Almost all kinds of RNA in eukaryotes undergo 3′ end processing. RNA 3′ end changes dynamically in composition and length, which determine the fate of modified RNA [1,2]. The 3′ end cleavage and polyadenylation in the nucleus is essential for general mRNA maturation in eukaryotes and canonical poly(A) polymerase (PAP) adds poly(A) tail to mRNA in a transcription-termination-coupled manner [3]. The synthesized poly(A) tails are covered by Poly(A)-binding proteins (PABs/PABPs). In addition to canonical PAP, the TENT family also acts on decoration of the RNA 3′ end through non-canonical tailing, such as uridylation, mixed tailing, as well as cytoplasmic polyadenylation et al. [4,5,6], thereby exerting multiple functions. According to substrate preference for ATP or UTP, eleven TENTs in the human genome are classified into two subfamilies, non-canonical poly(A) polymerase (ncPAP) and terminal uridylyltransferase (TUTase) (Table 1) [7]. The cytoplasmic polyadenylation event by TENT2 (GLD-2) enhances the stability and translation of particular mRNAs, who possess the cytoplasmic polyadenylation element (CPE), by extending their poly(A) tails in the processes of gametogenesis, embryogenesis and long-term memory [8,9,10,11,12,13,14]. Mono-uridylation or oligo-uridylation by TUTases including TENT1 (TUT1), TENT3A (TUT4), and TENT3B (TUT7) participates in the biogenesis and turnover of A-tailed mRNAs, histone mRNAs, microRNAs, and U6 snRNA et al. [15,16,17]. Interestingly, the distinct roles of TUT4/7-mediated mono-uridylation and oligo-uridylation are well illustrated in the case of the tumor suppressor let-7 microRNA family: oligo-uridylation of pre-let-7 promotes its decay and mono-uridylation of pre-let-7 affects its maturation [18,19,20,21,22,23]. Guanosine residues in poly(A) tail of mRNA, deposited through mixed tailing by TENT4A/4B, impede the deadenylase complex CCR4-NOT and enhance the mRNA stability [24,25]. It is known that eukaryotic mRNAs are degraded through multiple pathways and the major one is mediated by 3′–5′ exonucleases (deadenylases), such as CCR4–NOT and PAN2–PAN3 (Figure 1A) [26,27,28,29]. Guanosine insertions in poly(A) tails possibly disrupt their single-stranded A-form-like helix structure and hinder deadenylase function, thus reducing the degradation rates of the transcripts [30]. The function of G-content in mixed tailing has been revealed in mammals, *Arabidopsis*, and certain virus-infected cells [27,31,32,33].

Eukaryotic cells have evolved the RNA-based antiviral immunity to escape from viral infection. One well-known mechanism is that long double-stranded RNAs (dsRNAs) derived from the virus in infected cells induce RNA interference (RNAi) to specifically remove viral RNAs [34,35]. Dicer, a member of the RNase III family, cleaves these dsRNAs into virus-derived small interfering RNAs (viRNAs), which are loaded into Argonaute (AGO) proteins to form the RNA-induced silencing complex (RISC) and thereby silencing viral RNAs [36,37,38,39]. Recent studies have identified a novel mechanism of virus–host interaction, which is conserved across animals and mediated through 3′ tailing of viral RNAs [40,41,42]. Interestingly, two types of 3′ tails, U-rich tail and mixed tail, lead to distinct consequences. The U-rich tail deposited by TUTases at the viral RNA terminus triggers viral RNA decay [40,43,44]. On the contrary, some viruses employ mixed tails to protect their RNAs from decay [45]. Increasing evidence has suggested that the length and composition of the viral RNA tail are a hotspot for evolutionary battle between viruses and their hosts. 

Viral RNA 3′ tailing has emerged as an important target pathway for antiviral therapy and also the controlled mRNA 3′ tailing has great potential for improving RNA vaccine efficiency. Therefore, the comprehensive understanding is needed regarding the significance and action mechanism of RNA 3′ tailing in various organisms and virus infection. Here, we review biogenesis and function of mixed tail and U-rich tail particularly in the process of virus infection, and describe the impact of RNA 3′ tailing on virus–host survival competition and relevant antiviral therapy. 

## 2. Mixed Tail in Viral Infection

To date, guanosine residues in poly(A) tails have been found in human cells, *Arabidopsis*, viral infection, and embryos from several organisms including mouse, frog, *Drosophila*, and zebrafish [24,32,45]. Even though the research on RNA mixed tailing is still at an early stage, the enzymes responsible for the process have been illustrated in mammals, and the function and action mechanism have been revealed in mammals, *Arabidopsis*, and viral infection. The experimental data have suggested that host TENT4 is employed by hepatitis A virus (HAV), hepatitis B virus (HBV), and human cytomegalovirus (HCMV) to control viral RNA stability, and inhibitors targeting TENT4 have been rapidly developing as antiviral medicine.

### 2.1. The Cellular Function of Mixed Tail

TENT4A and TENT4B are predominantly responsible for generating mixed tail in mammals, decorating poly(A) tail with non-adenosine nucleotides, of which guanosine is the most common [24,42]. Because TENT4A/4B is mostly found in the nucleus, but also in the cytoplasm (Table 1) [46,47], so mixed tailing more likely occurs in the nucleus. It has shown that TENT4A/4B holds relaxed nucleotide selectivity during poly(A) tail synthesis and is more selective for GTP than UTP and CTP, and thus incorporates non-adenosine residues into poly(A) tail. Guanosine residues were mainly found at the positions close to 3′ ends of long A-tails (≥25 nt). Since a single guanosine residue is sufficient to impede the CCR4-NOT complex, the complex trims the tail until exposing the guanosine at the 3′ end (Figure 1B) [24,42]. As the result, mixed tailing, also known as G-content tailing, enhances mRNA stability by slowing down mRNA degradation. It needs further examination whether mixed tailing of mRNA has conserved biogenesis and function across vertebrates. 

Researchers have found that G-content in *Arabidopsis* poly(A) tails regulates translation efficiency but not mRNA stability of plant genes. Poly(A)-seq analysis of *Arabidopsis* has revealed that over 10% of poly(A) tails carry G-content, taking up 0.8–28% of each tail. Surprisingly, the data support that G-content in A-tail is negatively correlated with the binding efficiency of PABP on A-tail and further the gene with higher G-content has lower translation efficiency [31]. PABP is believed to identify the pure poly(A) primarily, and the binding of human PABP or yeast Pab1p to mRNA poly(A) tail requires 11–12 contiguous A-nucleotides [48,49,50]. Guanosine in poly(A) tail separates the tail into interspersed A-segments, supposed to reduce binding efficiency between PABP and A-tail and also translation efficiency of *Arabidopsis* genes (Figure 1C) [31]. The mixed tailing in mammals is generated by TENT4A/4B, whereas the producing mechanism in *Arabidopsis* is unclear. 

The different ways of mixed tailing in regulation of mammalian and plant genes indicate the possibility of its diverse action mechanism in varying organisms. Although its overall function has been illustrated in mammal and plant, many important questions are still remained. For example, it is unknown how the preferred nucleotide incorporation undergoes during mixed tailing and how particular genes have A-tails with high G-percentage. Interestingly, the clue to gene-specific increase in G-content in A-tail has come from studies of viral infection. 

### 2.2. The Pathological Function of Mixed Tail in Virus Infection

To survive from virus infection, hosts have evolved diverse antiviral immunity that does not solely rely on RNAi or an interferon pathway. Notably, RNA 3′ uridylation is an effective pathway for preventing viral invasion [51]. On the other hand, RNA 3′ mixed tailing is hijacked by viruses to stabilize their RNA and facilitate viral invasion [52]. Recent research indicated that RNA stability in HAV, HBV, and HCMV is closely related to TENT4A/4B. In addition, similar strategies are also used in several other viruses, including Norovirus, Saffold virus, and Kobuviruse, which indicates the active participation of TENT4A/4B in viral life cycle is a more general event [45,53,54,55]. 

**Figure 2:**
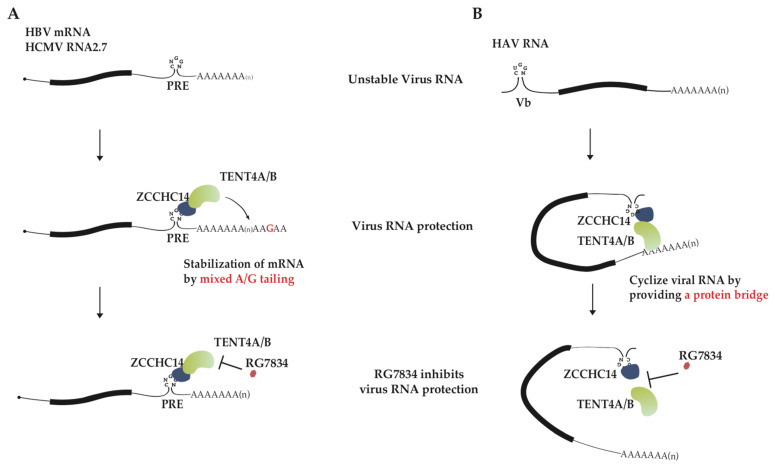
The proposed models for virus RNA stabilization by ZCCHC14-TENT4A/4B complex. (**A**) A model for inhibition of HCMV RNA2.7 and HBV mRNA stability by RG7834. TENT4A/4B is recruited by ZCCHC14 to the stem-loop of PRE in viral mRNA 3′ UTR, and thereby generating 3′ mixed tails at viral mRNA and disturbing CCR4-NOT-mediated RNA decay. RG7834 inhibits terminal nucleotidyltransferase activity of TENT4, destabilizing HBV and HCMV transcripts. (**B**) A model for RG7834 inhibition of HAV RNA synthesis. ZCCHC14 binds to stem-loop Vb in the HAV 5′ UTR and TENT4 might recognize the 3′ end of polyadenylated HAV genome RNA. ZCCHC14 and TENT4 interaction serves as a bridge that facilitates functional cyclization of the genome toward synthesizing its complementary RNA. RG7834 disrupts interaction between TENT4A/4B with ZCCHC14, interrupting genome cyclization and impeding genome RNA replication.

HBV and HCMV are double-stranded (ds) DNA viruses with the relaxed circular dsDNA and linear dsDNA as genomes, respectively. It had been assumed that mRNA maturation in the dsDNA viruses like HBV and HCMV might follow the processes similar to host, including 5′ capping, splicing and 3′ polyadenylation. Out of expectation, HCMV and HBV cleverly hijack host TENT4A/4B to produce mixed tail at the 3′ end of viral mRNA to facilitate their infection [45]. Remarkably, the process is well controlled and specific to viral transcripts with 3′UTR post-transcriptional regulatory element (PRE), and host RNA-binding protein (RBP) ZCCHC14 and enzyme TENT4A/4B coordinate to complete the process. The Smaug-like SAM domain in the zinc finger protein ZCCHC14 recognizes the CNGGN pentaloop in PRE. At the same time, ZCCHC14 attracts TENT4 and tethers it on viral mRNA to generate mixed tails. The non-templated addition of guanosines onto 3′ ends of HBV and HCMV mRNA enhances RNA stability by inhibiting degradation (Figure 2A). The pentaloop is present in almost all HBV mRNA species and HCMV RNA2.7, and their half-lives are significantly diminished when the enzyme TENT4A/4B is depleted [45]. The single or double knockout experiments of TENT4A/4B in HepG2 cells showed that two genes have redundant function, with TENT4B tailing HBV transcripts well when TENT4A is knocked out and TENT4A less capable of tailing when TENT4B is knocked out. In other words, TENT4B is primarily charged with stabilizing HBV mRNA at least in HepG2 cells [53]. Although this phenomenon might result from different expression levels of TENT4A and 4B in HepG2 cells, there is also another possibility. As ZCCHC14 was mainly observed in the cytoplasm, the different localization of TENT4A and 4B protein in cells led to the above phenomenon. 

HAV is a single-stranded (ss) RNA virus with a small positive-sense RNA as a genome. Viral genome RNA 3′ end is polyadenylated, but instead of 5′ capping, the 5′ end is covalently linked to a small viral protein VPg, the putative protein primer for minus-strand RNA synthesis. Although HAV replication cycle still remains unclear, its genome replication is expected to use RNA as template to directly synthesize complementary RNA [56]. HAV infection was also reported to require ZCCHC14 and TENT4A/4B [33,57]. However, unlike the case of HBV/HCMV, TENT4A/4B and ZCCHC14 mainly affect HAV RNA synthesis. Viral RNA poly(A) tail length, stability, and translation are unaffected but nascent viral RNA synthesis is significantly diminished by treatment of TENT4 inhibitor RG7834 [32]. ZCCHC14 could recognize a stem-loop with a CUGGN-type pentaloop in the 5′ UTR of viral genome RNA and recruit TENT4. A proposed model according to these data is that ZCCHC14-TENT4 forms a protein bridge between the 5′ UTR and 3′ end of viral genome RNA so circularizes HAV RNA and enhances viral RNA replication (Figure 2B) [32]. It remains to be determined whether HAV RNA replication is dependent on the terminal nucleotidyltransferase activity of TENT4.

In order to identify viral cis-acting elements playing roles in its RNA stability, translation and localization, high-throughput screening was conducted based on luciferase reporter system [55]. The 130 bp synthesized DNA segments of viral 3′ UTR origin were inserted into 3′ UTR of luciferase reporter for the screening and hundreds of elements were found from the experiment. Among them, Norovirus K3, Saffold virus K4, and Kobuviruse K5 elements were sensitive to TENT4 inhibitor RG7834 so all of them should be under the control of TENT4. Further comprehensive studies found that C-terminus and N-terminus of the ZCCHC2 protein interact, respectively, with K5 RNA element in 3′ UTR and TENT4 protein, thereby producing mixed tails, increasing mRNA stability and translation. Moreover, the function of K3 element is dependent on ZCCHC14 protein while K4 is insensitive to either ZCCHC14 or ZCCHC2, thus, the trans-acting factor for K4 needs further investigation [55]. 

The finding of TENT4A/4B as target proteins of antiviral small chemical RG7834 has greatly advanced the knowledge about the mechanism of viral gene expression [54]. Intriguingly, viruses from varying families with different genome types, sequences, and life cycles have evolved in a similar way, hiring host RBP-TENT4 complexes, to specifically stabilize viral RNA, which establishes a fantastic target pathway for developing generally workable antiviral medicine.

### 2.3. TENT4-ZCCHC14 and Anti-Hepatitis Virus Therapy

HBV infection is one of the most significant public health issues, with ~350 million chronic HBV patients worldwide [58,59,60]. Approximately 240 million patients with Chronic Hepatitis B (CHB) are Hepatitis B surface antigen (HbsAg) positive, exposed to the risk of cirrhosis and hepatocellular carcinoma (HCC) [61,62]. Unlike HBV, HAV infection usually causes acute hepatitis, ranging in severity from mild to severe [63,64]. In rare cases, a weakened immune system can make hepatitis A infection deadly.

Nucleotide analogues and immune modulators are widely-used antiviral agents that are effective in preventing the spread of infectious viruses, but these medicines have several disadvantages such as strong side effects and the development of drug resistance [65,66,67]. Currently, HBsAg has a major role in host immune escape and HBsAg together with HBV DNA levels are the hallmarks of chronic HBV infection, and HBsAg is the foundation for diagnosing infections, screening blood, and determining the cure for antiviral therapy [68,69,70]. So, HBsAg inhibitors have been extensively screened to overcome chronic HBV infection and they can be structurally divided into DHQ (dihydroquinazinone) and THP (tetrahydropyridine) classes [71,72]. 

RG7834, a small chemical in DHQ class, was developed by Roche as a HBsAg inhibitor, and could target HBV and reduce viral gene expression [73,74]. RG7834 eliminates viral antigens and DNA, having a distinct antiviral profile compared with nucleotide analogues. By reducing the viral components required to complete the virus life cycle as well as those involved in escaping the host immune system, RG7834 was believed to have the potential to inhibit HBV and improve HBV cure rates [75,76,77,78]. Indeed, oral treatment of HBV-infected humanized mice with RG7834 resulted in a 1.09 log reduction in HBsAg levels [73,74]. Meanwhile, oral RG7834 ingestion reduced HAV replication and profoundly interrupted the pathogenesis of animal models infected with HAV [32]. RG7834 was also evaluated for its safety in the first clinical trial with 49 participants, and no adverse reactions were reported (ClinicalTrials.gov NCT02604355). Unfortunately, subsequent clinical drug development failed due to its adverse neurotoxicity. Nevertheless, because recent studies have revealed TENT4A/4B as direct targets of RG7834, TENT4-ZCCHC module is currently emerging as a new therapeutic target for clinical drug development and presents a novel perspective on hepatitis virus therapy and chemoprevention. 

Roche has also released a series of THP class HBsAg inhibitors, among which, the representative compound 3 inhibits HBsAg and HBV DNA synthesis in HepG2.2.15 cells [73]. Li Zhang et al. synthesized THP HBsAg Inhibitor 17i, which exhibited the excellent in vitro anti-HBV potency with low toxicity, and dramatically reduced serum HBsAg and HBV DNA levels in HBV transgenic mice [79]. In conclusion, the discovery of new antiviral chemicals and therapeutic target pathways could coordinately accelerate drug development to achieve a functional cure for patients with hepatitis. 

## 3. U-Rich Tail in Antiviral Innate Immune Response

Viruses have evolved several ways in the RNA level to escape from host immune system whereas hosts have also developed multiple immune responses to resist virus invasion. Most organisms possess innate immune responses to recognize and eliminate viruses. Interferon, RNAi and RNA uridylation pathways function as natural antiviral defense mechanisms in various living creatures.

### 3.1. The Interferon and RNAi in Antiviral Immune Response

**Figure 3:**
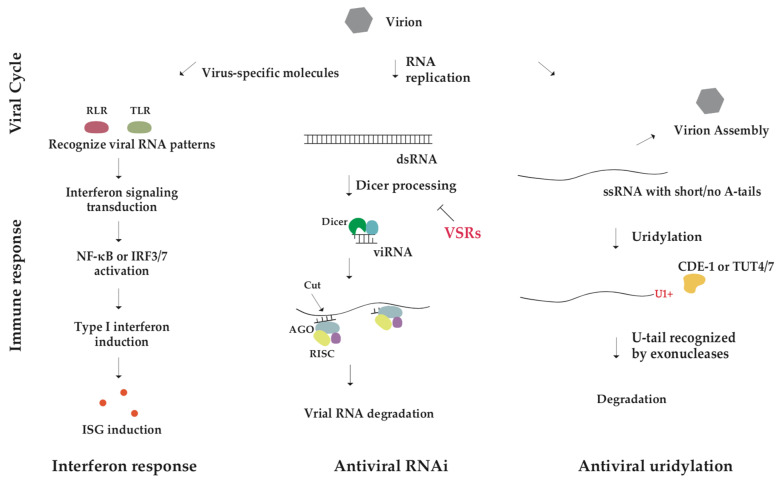
Overview of antiviral innate immune pathways. Interferon pathway (**left**), RNAi pathway (**middle**), terminal uridylation (**right**) resist virus infection. Viral RNA is uridylated by terminal uridylyltransferase CDE-1 in *C. elegans* and TUT4/7 in mammals. The U-tagged RNA undergoes a degradation pathway.

In mammals, cells detect viral infection by pattern recognition receptors (PRRs), and induce an interferon type I response in both cell-autonomous and non-autonomous manners [80]. Virus-specific molecules like 5′ tri-/di-phosphorylated RNA, ssRNA, and dsRNA are recognized by intracellular PRRs, mainly RIG-I-like receptors (RLRs) and Toll-like receptors (TLRs), activating interferon signaling transduction, which in turn triggers expression of type I interferons (IFNs) through the transcription factor NF-κB or IRF3/7-mediated pathway (Figure 3, the left panel) [80,81]. Produced interferons tune the surrounding cells to an antiviral state by inducing the expression of interferon-stimulated genes (ISGs), which include a variety of antiviral proteins. ISGs can further promote innate and adaptive immune responses against viruses [82,83]. 

In contrast, plants and invertebrates rely on powerful RNA interference (RNAi) to combat viral infection [84,85]. Dicer first processes viral dsRNA into virus-derived small interfering RNA (viRNA), and once the guide strand of viRNA is incorporated into AGO protein in the RNA-induced silencing complex (RISC), the complex uses viRNA to recognize and cleave viral RNA (Figure 3, the middle panel) [86]. Notably, some viruses encode viral suppressors of RNA silencing (VSRs) that can inhibit cellular RNAi pathway, so viral RNA can escape from degradation [87]. The antiviral role of RNAi in mammals has been widely debated, mainly because viRNAs, the hallmark of RNAi involvement in viral defense, are rarely detected in virus-infected mammalian cells. However, there are now several lines of evidence to support an important role of RNAi in mammalian antiviral response [88]. ViRNAs were observed in mammalian embryonic stem cells infected by encephalomyocarditis virus (EMCV) or Nodamura virus (NoV). Undifferentiated stem cells only express a reduced level of interferon [87]. ViRNAs were also accumulated in suckling mice and cultured hamster cells infected by mutant NoV lacking the RNAi suppressor protein B2, but not wild type NoV [89]. Thus, ViRNAs could be observed only in mammalian cells with a less effective interferon system and/or infected by a virus without VSRs. In general, differentiated mammalian cells depend on the interferon response, whereas undifferentiated stem cells can utilize RNAi for defense against viruses.

### 3.2. U-Rich Tail in Antiviral Immune Response

Interestingly, uridylation has been demonstrated as a conserved host innate immune response against viral infection. The screening of immunodeficient mutants in OrV (Orsay virus)-infected *C. elegans* identified an essential role of CDE-1 in resisting viral infestation. CDE-1, a homolog of mammalian TUT4/7, uridylates the 3′ end of the OrV RNA genome and drives its degradation independent on the RNAi pathway (Figure 3, the right panel) [40]. OrV possesses two positive-sense RNAs as its genome, and adding mono-U in its genome RNAs by CDE-1 results in the dimer-U termination of the genome, which triggers RNA degradation [90,91]. Furthermore, studies in mouse embryonic fibroblast (MEF) cells infected with influenza A virus (IAV), a negative-sense ssRNA virus, revealed that non-templated U-tail was added to IAV mRNA terminus by TUT4/7 and that the oligo-U tail (more than two U) was the most common. TUT4/7 knockout resulted in increased IAV mRNA and protein. Uridylation acts as a shield through reducing viral expression and infection [40].

Host TUT4/7-mediated uridylation was also recently reported to delay mouse hepatitis virus (MHV) replication in mouse 17-CL1 cells [44]. MHV, a positive-sense ssRNA virus, belongs to the Coronavirus family and both its genome and subgenomic RNAs (sgRNAs) have poly(A) tails. About 9% of MHV RNAs have uridylated termini, which are mainly divided into two pools: one with ~44 nt long poly(A) tails and the other with shorter than ~22 nt poly(A) tails. TUT4/7 seemed to be only responsible for the uridylation of subgenomic RNAs with tails shorter than 22 nt. Remarkably, depletion of TUT4/7 increased viral RNA load, thus, it was proposed that TUT4/7 uridylates MHV subgenomic RNAs for degradation and thereby delaying viral replication. 

The 3′ uridylation was also detected in viral RNAs from varying plant viruses. The extensive profiling of uridylation was carried out using 3′ RACE-seq for the representative ~20 plant viruses with positive-sense ssRNA genomes and uridylation was present in all 47 viral RNAs investigated. According to knockout experiments in *Arabidopsis*, both TUTases of HESO1 and URT1 participate in the uridylation of viral RNAs from turnip crinkle virus (TCV) and turnip mosaic virus (TuMV). However, the double knockout of TUTases did not affect viral infection [92]. Another study also found non-templated U-rich tails in viral RNAs from mycoviruses, plant viruses, and animal viruses although the results might have some bias because cDNA synthesis in the study was primed with an oligo(dA)_18_ primer [41]. Additionally, TUT4/7 adds U-tails to the LINE-1 mRNA and inhibits its retrotransposition to establish mammalian host genome stability [93,94].

Taken together, uridylation appears to be wide-spread in eukaryotic viruses. Although it is not fully clear, TUTases are more likely to attack viral RNA that lacks or with short A-tail, which may not be protected and is critical for host recognition of pathogens [92,95,96,97]. Consequently, uridylation promotes viral RNA degradation and protects host cells from viral invasion in some cases. Thus, it is evident that the 3′ end is crucial to both the invader and host for survival competition [51,98]. In the future, the research should concentrate on the function and action mechanism of TENTs in various virus–host models to provide new concept for antiviral medication.

## 4. Conclusions and Discussion

In eukaryotes, RNA tailing is often associated with RNA trimming or decay. In the last 30 years, there have been remarkable breakthroughs in understanding RNA uridylation, a conserved post-transcriptional gene regulation mechanism with a wide range of RNA substrates in living organisms, as an essential tool for intracellular RNA monitoring. New attention has been paid to mixed A/G tailing to date. Recent studies have been focused more on the substrate selectivity and biochemical function of TENT4. TENT4A and TENT4B are two human homologues of the yeast Trf4p protein. In yeast, the Trf4p–Air2p–Mtr4p polyadenylation (TRAMP) complex promotes nuclear surveillance of aberrant mRNAs, rRNAs, snRNAs, snoRNAs and tRNAs [99,100,101,102]. A TRAMP-like complex consisting of TENT4B, ZCCHC7 (Air1/2 homologue) and RNA helicase MTR4 is present in mammalian cells [46,103,104]. The phenomenon of TENT4A/4B producing mixed A/G tailing has been illustrated more recently and is very dissimilar to the function of TENT4A/4B to eliminate abnormal RNAs previously. In mammals, TENT4 produces mixed tailing, which disturbs CCR4-NOT complex and protects mRNA from degradation. Mixed A/G tailing has also been found in *Arabidopsis*. Further research needs to answer whether mixed A/G tailing is conserved in varying organisms, whether its substrates are ubiquitous or specific, as well as the underlying mechanism of its substrate selection.

The study on RNA non-A tailing illustrates the novel mechanism of virus–host interaction. Modification in viral RNA bypasses or stimulates the host machinery for RNA degradation and thus influencing infection success. The widespread presence of 3′ uridylation in eukaryotic RNA viruses suggests the uridylation-directed RNA decay pathway as a universal defense system against viruses. Perhaps in response to this threat, some viruses have evolved to modify the 3′ ends of their RNAs, which protects them against host degradation, like the mRNAs of HBV and HCMV with mixed tails and single-stranded RNAs of *Flaviviridae* with highly structured 3′ ends [105,106,107]. Tail modifications may also be used to regulate the activity of other transposons. RNAi targets and transposon RNAs are modified by a *C. elegans* poly(UG) polymerase MUT-2 by adding p(UG) tails. With over 16 nt perfectly alternating U and G nucleotides, RNA fragments attached to the p(UG) tail can act as gene-silencing agents that suppress gene expression [108,109]. In addition to tailing, another RNA modification N6-methyladenosine (m^6^A) has been recently discovered to have a role in the life cycles of many viruses as well as in cellular response to viral infection [110,111,112]. Parasite–host interactions are also affected by polyadenylation [98]. The fact is fascinating that viruses with varying life cycles and tissue specificities have evolved similar strategies to interfere with host defense. According to this convergent evolutionary nature, multiple viruses may possess similar tail modification mechanisms, perhaps having learned to avoid host RNA degradation pathways or partially inactivating host degradation pathways.

The involvement of TENT and its cofactor in viral life cycles may provide mechanistic insights into the development of a new category of antiviral drugs. In spite of requiring further investigation, the role of RNA tailing as an essential regulatory instrument will provide an unexpected opportunity for antiviral treatment and chemoprevention.

## Figures and Tables

**Table 1:** The RNA substrates and localization of human TENTs.

Subfamily	Enzyme (Synonyms)	RNA Substrate	Activity	Localization
TENT1	TUT1 (U6 TUTase, PAPD2, RBM21, URLC6, STARPAP)	U6 snRNA Pre-mRNA	oligouridylation polyadenylation	nucleolus nuclear speckle nucleoplasm cytosol mitochondrion
TENT2	TENT2 (GLD-2, PAPD4, TUT2, APD4)	mRNA miRNA	monoadenylation oligoadenylation polyadenylation	part of nuclear RNA-directed RNA polymerase complex cytoplasm
TENT3	TUT4 (PAPD3, TENT3A, ZCCHC11)	mRNA Histone mRNA LINE-1 mRNA Pre-miRNA miRNA Viral RNA Pre-rRNA Pol III-ncRNA TSS RNA	monouridylation oligouridylation	nucleolus cytosol cytoplasm cytoplasmic ribonucleoprotein granule extracellular space extracellular exosome
	TUT7 (PAPD6, TENT3B, ZCCHC6)			nucleoplasm cytosol cytoplasm
TENT4	TENT4A (PAPD7, TUT5, TRF4-1, LAK1, POLK, POLS)	mRNA Viral RNA	polyadenylation mixed tailing	nucleus nucleoplasm nuclear membrane nucleolus Golgi apparatus part of TRAMP complex
	TENT4B (PAPD5, TUT3, TRF4-2)	mRNA Viral RNA miRNA Pre-rRNA rRNA snoRNA scaRNA Y RNA hTR	monoadenylation oligoadenylation polyadenylation mixed tailing	nucleolus plasma membrane cytosol cytoplasm part of TRAMP complex
TENT5	TENT5A (OI18, XTP11, FAM46A, C6orf37)	mRNA	polyadenylation	nucleus cytoplasm
	TENT5B (FAM46B)			nucleus cytoplasm
	TENT5C (FAM46C)			nucleus nucleoplasm cytoplasm centrosome
	TENT5D (CT112, CT1.26, FAM46D)			nucleus cytoplasm
TENT6	MTPAP (PAPD1, TENT6, SPAX4)	MT-mRNA MT-tRNA	oligoadenylation polyadenylation	nucleoplasm mitochondrion intracellular membrane-bounded organelle
